# An mHealth App for Decision-Making Support in Wound Dressing Selection (WounDS): Protocol for a User-Centered Feasibility Study

**DOI:** 10.2196/resprot.9116

**Published:** 2018-04-24

**Authors:** Scott Jordan, Jane McSwiggan, Joanne Parker, Gayle A Halas, Marcia Friesen

**Affiliations:** ^1^ Department of Electrical & Computer Engineering University of Manitoba Winnipeg, MB Canada; ^2^ Wound Care, Nursing Initiatives Winnipeg Regional Health Authority Winnipeg, MB Canada; ^3^ Department of Family Medicine University of Manitoba Winnipeg, MB Canada

**Keywords:** mHealth, wounds, wound dressing, wound management

## Abstract

**Background:**

Primary care health professionals, especially family physicians, see a variety of wounds, and yet—despite the frequency of providing wound care—many family physicians do not feel confident in wound care management. This is partly due to a lack of formal wound education in Family Medicine programs. While there are numerous electronic wound care resources available in the UK and North America, none were identified that address the specific need in supporting clinical decision-making in wound dressing selection. At the same time, healthcare providers are increasingly using technology in personal and professional contexts, and a logical extension is to use technology for knowledge translation strategies.

**Objective:**

This work developed a prototype mobile health software application named *WounDS*, designed to support clinical decision-making in selecting wound dressings. This article presents the development and evaluation plan for the *WounDS* app.

**Methods:**

*WounDS* has been developed on the iOS platform. The primary specification included ease of use, in that one of the primary influences in user adoption would be the ability to receive a wound dressing recommendation in under 30 seconds and under 5 taps on the screen. The *WounDS* app guides users through a series of binary decisions for assessing the wound and provides a wound dressing recommendation. The selection algorithm is based in best practices using the Wound Bed Preparation Paradigm.

**Results:**

Current work is underway to examine the implementation needs for *WounDS* to be most effectively utilized and to pilot test its feasibility and use in clinical care. Data will be collected through user trials, focus groups, and user metadata will be collected within the app. Optimizing these preconditions will enable a subsequent phase of study to determine effects on clinical decision-making and clinical outcomes.

**Conclusions:**

*WounDS* is designed for knowledge translation, use of technology in clinical decision-making, and continuity of care. The benefits of *WounDS* include the potential to improve healthcare providers’ competency in wound management and to improve wound healing through better alignment with evidence-based best practices in wound dressing selection, consistency in care from primary to community care, and subsequent downstream impacts in quality of life for patients.

## Introduction

This work developed a mobile health (mHealth) software application on the iOS platform, designed as a guide for selecting wound dressings that are maximally aligned with the patient’s wound assessment, care plan, and best practice in wound healing—in short, a *Woun*d *D*ressing *S*election app (*WounDS*, or “the app”). *WounDS*, designed for iPhone and iPad, is currently a functional, stand-alone prototype app. It is designed to support (but not replace) clinical decision-making in wound dressing selections, particularly for healthcare providers with little education or experience in wound management. Current work is focused on evaluation of the app’s use, and this article presents the development and evaluation plan for the *WounDS* app.

Primary care health professionals, especially family physicians, see a variety of wounds in their practices. These include skin abrasions, burns, lacerations secondary to trauma, leg ulcers, diabetic foot ulcers, and less commonly, pressure injuries. Family physicians can best serve their patients if they have access to current and comprehensive knowledge and skills in wound management [1-6]. Yet, family physicians do not feel confident in wound care management [7]. Currently, undergraduate medical students in Manitoba, Canada receive no formal wound care education in medical school. Family Medicine residents preparing to go into practice as family physicians receive only limited formal education on wound care, which includes up to 3 hours of content on differentiating wounds, causes of wounds, wound healing principles, and choosing the appropriate dressing for various types of wounds. The accreditation standards used by the College of Family Physicians do not include wound care education training as a curriculum requirement for Family Medicine residents [8].

An Ontario study (n=214) reported that only 16% (34/214) of family physicians felt confident in their ability to manage leg ulcers, 61% (130/214) did not feel they knew enough about wound care products, and more than 50% (107/214) were unaware of the use of compression as an effective treatment for venous ulcers [7]. Further, a national roundtable reported that appropriate dressing selection and use were identified for only 20% of wounds [9]. These findings supported the need for better guidance in wound management and dressing selection for decreased healing time, returning patients to optimal functioning sooner, and improved quality of life.

Little published data exists in Canada on the exact cost of wound care, although estimates are that wound care amounts to Can $3.9 billion per year in costs to the Canadian health care system [9]. An Ontario study estimates that lower limb ulcers alone cost Can $100 million per year [7]. More than 80% of chronic wounds such as leg ulcers occur in the community, and family physicians working in primary care are often patients’ first contact for treatment. Chronic wounds are expected to become an increased economic burden given an aging population and co-morbid conditions such as diabetes and obesity [5,7]. The cost of wound care includes dressings and other materials, clinician time and hospitalization. Optimal wound management, from treatment to healing (if possible) requires careful assessment of the cause of the wound, person-centered concerns such as pain, and each wound’s unique characteristics. When an advanced wound care dressing with a longer wear time is selected, the benefits outweigh the initial dressing costs, by having fewer dressing changes, maintaining an even temperature, reducing the exposure to contaminants, and reducing labor costs [10,11].

In healthcare delivery in Manitoba, Canada, wound care and wound management decisions may be made by both nurses and physicians, with nurses providing care in home care settings as well as clinics. When Family Medicine residents provide wound care in teaching clinics, they usually consult with on-site nurses with wound care skill sets to assist them in determining appropriate wound dressings for patients presenting to the clinic. Yet, not all teaching sites used in the Family Medicine residency program employ nurses; thus, wound care is then determined by the Family Medicine resident and the supervising physicians, who also often have limited wound dressing selection knowledge. In such cases, the wound care products may be selected on the basis of a practitioner’s familiarity, preference and ease of use. Making informed, individualized wound dressing decisions based on best practices occurs less frequently despite resources and evidence-informed tools being available. On a practical level, it can be overwhelming for Family Medicine residents to evaluate the categories of wound care products for use, resulting in the default choices to the most familiar products.

There are numerous wound care resources available in the UK and North America, but we are not aware of any that address this specific need in supporting clinical decision-making in wound dressing selection. Currently, posters and other wound dressing product information (often from proprietary sources) exist to help guide in dressing selections. However, practitioners have indicated that adding these resources to busy units is a form of white noise. Concomitantly, there is an increasing emphasis on electronic communication in wound management to improve the efficiency of care, the patient and caregiver experience, and ultimately the clinical outcomes. Electronic Health (eHealth) and mHealth initiatives in wound care are conjectured to assist in prevention and treatment by facilitating different types of healthcare interventions, changing user behaviors, enhancing communication between patients and providers, and providing education [12-15].

Healthcare providers are increasingly using technology in personal and professional contexts, and a logical extension is to use technology for knowledge translation strategies, rather than continuing to rely on strategies that have not led to proven outcomes. There are several wound assessment apps on the market, including SmartWoundCare [16] (mobile app for handheld devices), How2Trak [17] (wound care software on a web-based interface), WoundRounds [18] (mobile app for handheld devices), and relative newcomers WoundMAP pump, Ulcercare, and Wound Mender in various stages of development [19]. These apps are all focused on assessing and documenting the wound, and none incorporate wound dressing selections. In areas outside of wound care, mobile consumer devices are increasingly capable of meaningful applications in mHealth, such as apps that range from allowing users to track diet and fitness, health condition monitoring (eg, diabetes [20]; arthritis [21]), and the use of mobile devices to replace paper records and share information between healthcare providers (eg, [22-24]).

Within this context, *WounDS* was developed for primary care family physicians as well as other healthcare providers (eg, registered nurses (RNs), nurse practitioners, clinical nurse specialists and MDs) delivering wound care in tertiary- and long-term care facilities as well as community settings. *WounDS* is designed to support (but not replace) clinical decision-making by serving as a tool to update best practices in wound care. A healthcare provider may rely on it more heavily in the early stages of their training and practice, and over time they may use it to confirm decisions they reach based on repeated exposure to wounds and their concomitant accumulated knowledge and experience. For Family Medicine residents with little wound care education, *WounDS* can assist in developing competency in sound wound dressing selection over time, particularly in the absence of a nurse’s or staff physician’s expertise.

## Methods

*WounDS* was developed to a functional prototype app on the iOS platform by an interdisciplinary development team with expertise in academic research and clinical practice in fields such as Nursing, Occupational Therapy, Wound Care, Computer Engineering, Biomedical Materials, and Family Medicine Research.

The *WounDS* app was designed using Xcode, Apple Inc’s integrated development environment (IDE) for developing software for macOS, iOS, watchOS, and tvOS. The *WounDS* app uses Apple’s Cocoa Touch software framework, which is the application programming interface (API) used for the iOS, watchOS, and tvOS operating systems. The language used to develop the *WounDS* app is Swift 3.0, the most current version of Apple’s alternative to the Objective-C language. Swift 3.0 is designed to work cohesively with Apple’s Cocoa Touch software framework and is included in Xcode. The source code is available from the authors and will be made available in GitHub.

*WounDS* was designed for task-technology fit, which asserts that a technology will be used and will have a positive impact on performance if its capabilities match the tasks to be performed. A number of factors contribute to task-technology fit. In *WounDS*, the quality and reliability of the app are facilitated by maintaining a small and simple software architecture and an uncomplicated user interface. The app is stored on the user’s mobile device (phone or tablet) and is not server-based. This facilitates its accessibility and self-directed authorizations by the user. To support timeliness, updates to the app will be based on user reviews and feedbacks, as well as product changes in wound dressings used by the regional health authority. These updates will be available in the same way as app updates are available through the iOS App Store.

A significant focus has been placed on ease of use as a factor of task-technology fit. The conjecture is that one of the primary influences in user adoption would be the ability to receive a wound dressing recommendation in under 30 seconds and under 5 taps on the screen. This reflects a “lazy user model” in which a user will select a solution (eg, *WounDS*) from within a set of solutions (eg, *WounDS*, internet look-up, posters on the wall, etc) based on the amount of effort required. These targets (30 seconds and 5 taps) reflect what Family Medicine residents have reported for a similar app that provides decision support for pre-operative checklists in the regional health authority, relative to its appeal and likelihood of use in clinical practice. In general website navigation, the “3-click rule” assumes that users will become frustrated if they cannot find desired information in 3 clicks. In this work, 5 clicks (taps) is considered acceptable given the distinct difference between general internet browsing with no certainty that the desired information will be located, in contrast to the use of a tool in clinical practice for specific purposes and with certainty that a response will be available.

The *WounDS* app guides users through a series of binary decisions for assessing the wound, to limit the wound dressing options to those that are best aligned with the individual care plan, including both generic and proprietary options. Furthermore, it limits the options to those with which a health region may have purchasing contracts. Using preset options related to the health region’s purchasing contracts is another factor of task-technology fit. The app is not associated with any patient per se, but rather serves as a deductive selection tool, akin to finding the correct recipe for something. *WounDS* will consider financial efficiencies when making suggestions, eg, less expensive dressings changed daily vs more expensive dressings that can stay on for multiple days. Integrated support features also increase the functionality and ultimate applicability of the *WounDS* app to others. These support features include help screens, glossary, links to external resources, and key salient content regarding wound management and the principles of wound dressing selection.

The selection algorithm is based on best practices using the Wound Bed Preparation Paradigm [11], as part of an overall patient-centered wound care approach which aims to treat the cause, treat the wound, and treat the patient’s concerns (eg, pain). The healthcare provider considers wound type and status, size and colour, location, duration, skin and other tissue characteristics, moisture balance, infection and inflammation, and wound edges as the complex determinants for an individualized care plan which includes wound dressings. For example, in the area of tissue alone, practitioners assess the epithelium, granulation, exposed tissues (bone, muscle, or tendon), eschar or slough, and infection.

Other assessments towards wound dressing selection include wound temperature, moisture balance, exudate, and pain associated with a wound. There are numerous types of wound dressings, including but not limited to, acrylic, antimicrobial, foam, hydrocolloid, hydrofibre, hydrogel, and textile. This context demonstrates that there are dozens of possible interactions between dozens of parameters associated with the wound and with a particular dressing, and the *WounDS* app supports clinical decision-making towards an optimum selection for the individual’s care plan.

**Figure 1:**
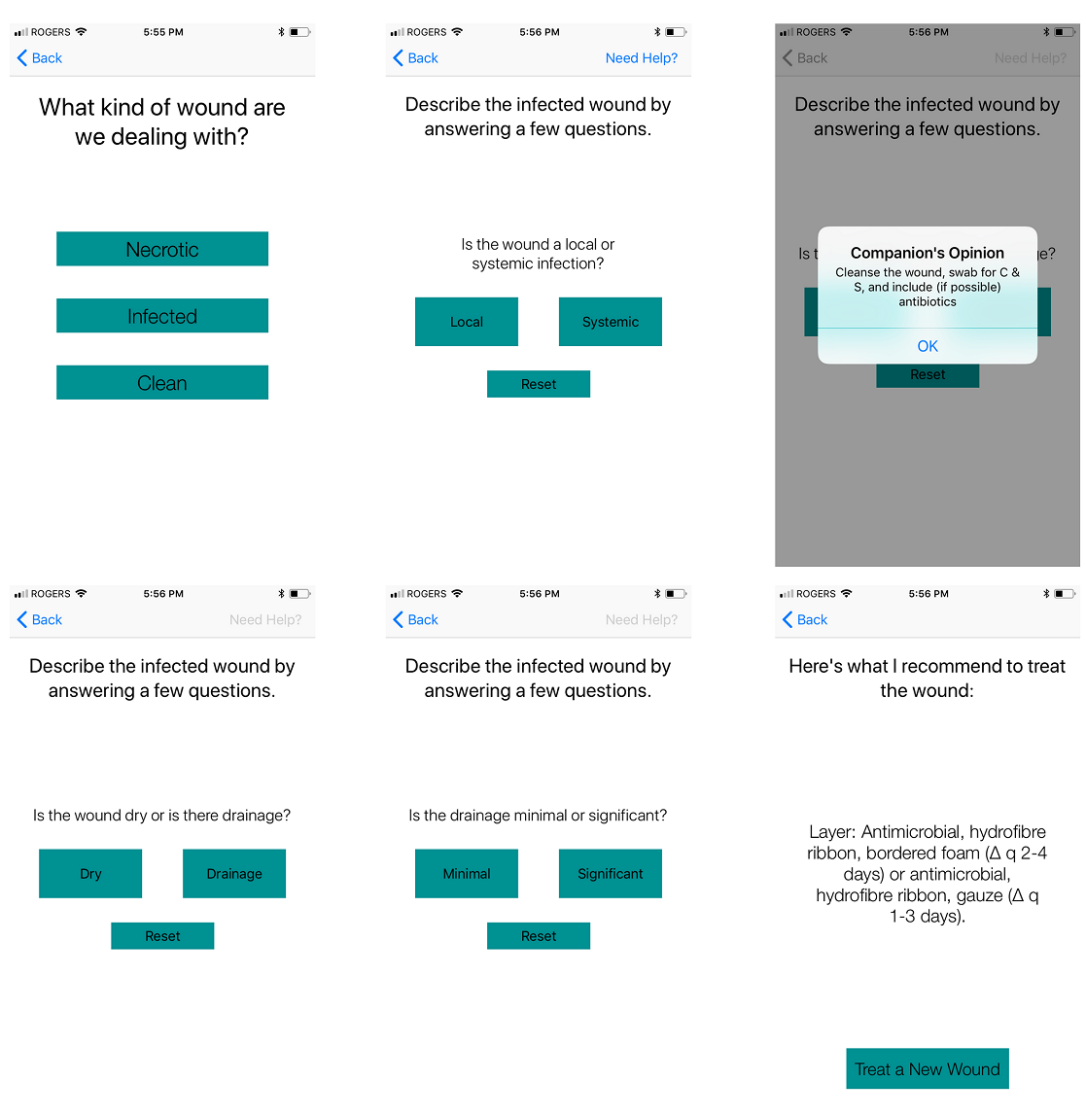
Sample user response pathway: infected - systemic - (okay) - drainage - significant - recommendation.

**Figure 2:**
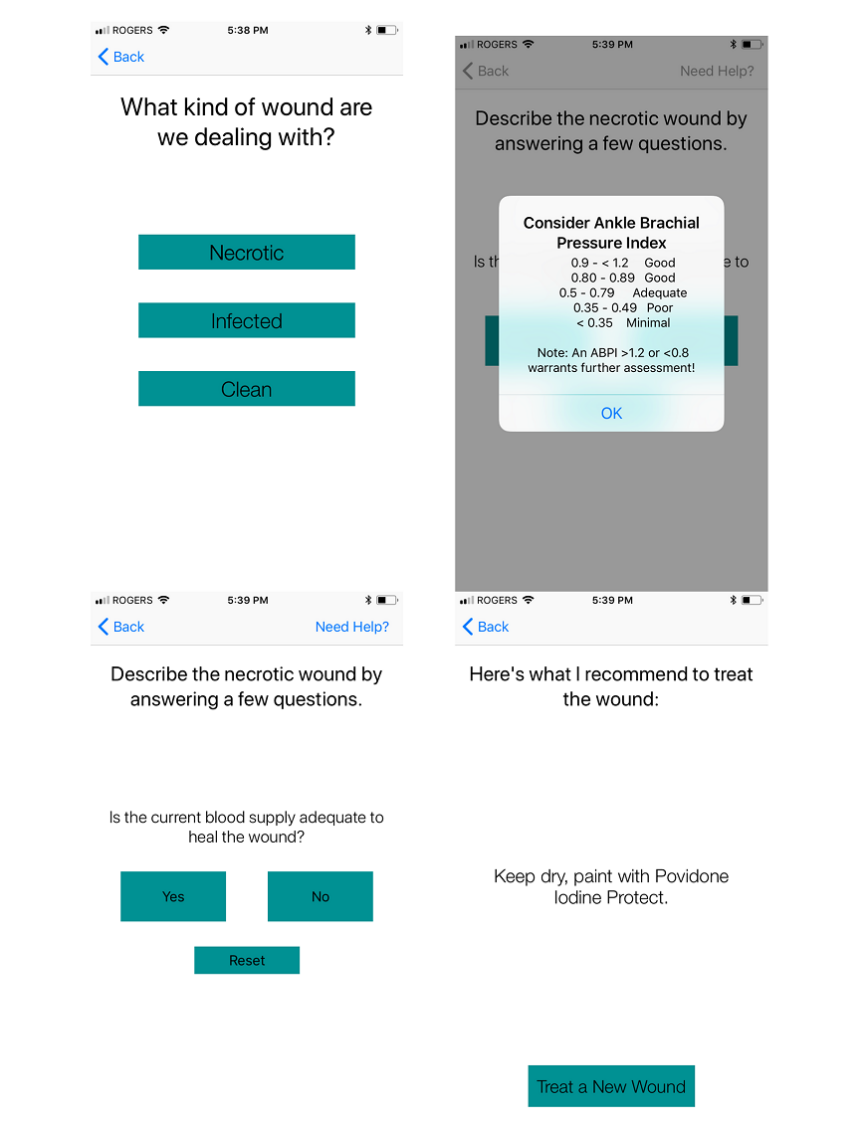
Sample user response pathway: necrotic - (okay) - no - recommendation.

Figures 1 and 2 display two representative pathways through the *WounDS* app. A key feature is that the user should be able to receive a recommendation in less than 30 seconds or 5 taps, for the app to be useful in day to day practice.

## Results

Currently, a study is underway to examine the implementation needs for *WounDS* to be most effectively utilized and to pilot test its feasibility and use in clinical care. Optimizing these pre-conditions will enable a subsequent phase of study to determine effects on clinical decision-making and clinical outcomes. Upon receipt of research ethics approval from the collaborating institutions, two phases will be undertaken.

In Phase 1, a qualitative usability study, design feedback from a focus group with Family Medicine residents and preceptors, including family physicians, nurses and nurse practitioners will be collected. Four case studies will serve as a basis upon which to examine the use of *WounDS* in a simulated context. The focus group participants will provide feedback to refine the app design, specifically advising on content, look-and-feel, and app functionality. They will also contribute to the design of an app support kit to accompany the use of *WounDS* and provide background information for the decision-making algorithm of *WounDS*. The Family Medicine residents are in an ideal position to provide feedback as they are generally high users of apps (thus providing a good comparison of *WounDS* usability and user interface to other apps). They will be asked to share their challenges in dressing selection following initial wound assessment. The preceptors are very involved in educating residents about wound management and will have valuable insights and suggestions for use among novice physicians as well as their own perspectives as a more experienced cohort of clinicians.

Phase 2 will involve implementing *WounDS* among a sample of 15 users (Family Medicine residents and home care nurses)—a sample size consistent with similarly designed studies [25-27] and appropriate for a purposeful sampling approach with the population of target users at the participating institutions. The users will be provided with the app support kit as part of their training for wound assessment and management and an introductory face-to-face training session. Users will be asked to trial *WounDS* in practice for up to four months. This timeframe was chosen to generate data on clinical utility quickly enough to capitalize on initial impressions and make changes. At the same time, the timeframe acknowledges that while home care nurses may see wounds on a near-daily basis, Family Medicine residents may not see wounds consistently or frequently, and a four-month timeframe will provide the opportunity for them to use the tool repeatedly. It is noteworthy that the infrequent presentation of wounds is exactly why the app is anticipated to be useful to Family Medicine residents, in that it supports information they do not use on a daily basis.

Following use in practice, the users will be invited to a focus group to determine whether and how the app was integrated into practice workflow, ease of use, and efficiency in helping to make best practice wound management decisions for various wound types. Members of the research team will be able to directly observe Family Medicine residents and document their use of *WounDS* in direct patient care. Their clinical expertise and in-depth familiarity with the clinical context will enable a rich textual narrative regarding the influence of implementation factors such as fit, how the app was used in practice, and its ability to be integrated into clinical flow.

In addition, *WounDS* will be designed to collect and store metadata from app use to gain a better understanding of users’ navigation pathways and movement through the algorithm, number of times users logged into the app, and what information was provided. Using a unique identifier, we will be able to compare the app-facilitated dressing selection to actual dressing selection as indicated in the patient’s medical record. This process of linking selections in the pilot phase will determine its research effectiveness for the larger subsequent study assessing service and client outcomes.

Both phases will provide an opportunity to address implementation issues as well as inform data collection for a subsequent clinical trial to examine patient outcomes.

## Discussion

*WounDS* is designed for knowledge translation, use of technology in clinical decision-making, and continuity of care. The benefits of *WounDS* include the potential to improve wound healing through better alignment with evidence-based best practices in wound dressing selection, consistency in care from primary to community care, and subsequent downstream impacts in quality of life for patients. Furthermore, *WounDS* can enhance healthcare providers’ capacity to deliver wound care and can enhance wound care knowledge transfer among healthcare providers and can potentially lead to cost savings for the health region. Current progress has resulted in a functioning prototype and an evaluation study in progress. It is noteworthy that Family Medicine residents are keen to engage with wound care specialists on this initiative.

*WounDS* is also the first known mHealth app of its type for wound dressing selection and it will serve as a proof-of-concept for this particular application. There are possible extensions for this concept that include integration with electronic medical record systems and integration with similar technology-based decision systems into other areas of clinical care. The latter could include assessment and treatment of specific wounds (pressure ulcers, diabetic foot ulcers) via SmartWoundCare [16], also developed within the research team, as well as blood glucose monitoring, blood pressure monitoring, and other self-monitoring tools.

## Abbreviations

WounDS: Wound Dressing Selection prototype mobile app

## References

[1] Lees TA, Lambert D (1992). Prevalence of lower limb ulceration in an urban health district. Br J Surg.

[2] Nelzén O, Bergqvist D, Lindhagen A, Hallböök T (1991). Chronic leg ulcers: an underestimated problem in primary health care among elderly patients. J Epidemiol Community Health.

[3] Simon DA, McCollum CN (1996). Approaches to venous leg ulcer care within the community: compression, pinch skin grafts and simple venous surgery. Ostomy Wound Manage.

[4] McGuckin M, Kerstein MD (1998). Venous leg ulcers and the family physician. Adv Wound Care.

[5] Sen C, Gordillo G, Roy S, Kirsner Robert, Lambert Lynn, Hunt Thomas K, Gottrup Finn, Gurtner Geoffrey C, Longaker Michael T (2009). Human skin wounds: a major and snowballing threat to public health and the economy. Wound Repair Regen.

[6] Little SH, Menawat SS, Worzniak M, Fetters MD (2013). Teaching wound care to family medicine residents on a wound care service. Adv Med Educ Pract.

[7] Graham ID, Harrison MB, Shafey M, Keast D (2003). Knowledge and attitudes regarding care of leg ulcers. Survey of family physicians. Can Fam Physician.

[8] The College of Family Physicians of Canada The College of Family Physicians of Canada.

[9] Ahearn P, Gardner P, Harley C, Latocki M, Paquette F, Stone J, Teague L (2012). The perfect storm: Summary of a national wound care stakeholder round table. Wound Care Canada.

[10] Bryan J (2004). Moist wound healing: A concept that changed our practice. Journal of Wound Care.

[11] Sibbald R, Goodman L, Woo K, Krasner DL, Smart H, Tariq G, Ayello EA, Burrell RE, Keast DH, Mayer D, Norton L, Salcido RS (2011). Special considerations in wound bed preparation 2011: An update. Advances in Skin and Wound Care.

[12] Friesen MR, Hamel C, McLeod RD (2013). A mHealth application for chronic wound care: findings of a user trial. Int J Environ Res Public Health.

[13] White PJF, Podaima BW, Friesen MR (2014). Algorithms for smartphone and tablet image analysis for healthcare applications. IEEE Access.

[14] Kratzke C, Cox C (2012). Smartphone technology and apps: Rapidly changing health promotion. Int Electronic J of Health Educ.

[15] Berry D, Blumenstein B, Halpenny B, Wolpin S, Fann JR, Austin-Seymour M, Bush N, Karras BT, Lober WB, McCorkle R (2011). Enhancing patient-provider communication with the electronic self-report assessment for cancer: A randomized trial. J Clinical Oncology.

[16] (2018). SmartWoundCare.

[17] Health Outcomes Worldwide.

[18] WoundRounds.

[19] (2013). Mobihealth News.

[20] Cafazzo JA, Casselman M, Hamming N, Katzman DK, Palmert MR (2012). Design of an mHealth app for the self-management of adolescent type 1 diabetes: a pilot study. J Med Internet Res.

[21] Georgia Tech GVU Centre.

[22] Health Outcomes Worldwide.

[23] Olawale D, Ferens K, Griffith B, Podaima B (2015). A smart shoe to prevent and manage diabetic foot diseases.

[24] Halas G, Katz A, Dean J (2010). Computer-based risk assessment: Evaluating use in primary care. Electronic Healthcare.

[25] Zhang M, Ho R, Hawa R, Sockalingam S (2016). Analysis of the information quality of bariatric surgery smartphone applications using the silberg scale. Obes Surg.

[26] Man C, Nguyen C, Lin S (2014). Effectiveness of a smartphone app for guiding antidepressant drug selection. Fam Med.

[27] Brown B, Cheraghi-Sohi S, Jaki T, Su T, Buchan I, Sperrin M (2016). Understanding clinical prediction models as 'innovations': a mixed methods study in UK family practice. BMC Med Inform Decis Mak.

